# Heterocycles as a Peptidomimetic Scaffold: Solid-Phase Synthesis Strategies

**DOI:** 10.3390/ph14050449

**Published:** 2021-05-10

**Authors:** Aizhan Abdildinova, Mark J. Kurth, Young-Dae Gong

**Affiliations:** 1 Innovative Drug Library Research Center, Department of Chemistry, College of Science, Dongguk University, 26, 3-ga, Pil-dong, Jung-gu, Seoul 04620, Korea; aizhik.a91@gmail.com; 2 Department of Chemistry, University of California, Davis, CA 95616, USA

**Keywords:** solid-phase synthesis, peptidomimetics, peptidomimetic synthesis, protein secondary structure mimetics, solid-phase peptide synthesis strategies

## Abstract

Peptidomimetics are a privileged class of pharmacophores that exhibit improved physicochemical and biological properties. Solid-phase synthesis is a powerful tool for gaining rapid access to libraries of molecules from small molecules to biopolymers and also is widely used for the synthesis of peptidomimetics. Small molecules including heterocycles serve as a core for hundreds of drugs, including peptidomimetic molecules. This review covers solid-phase synthesis strategies for peptidomimetics molecules based on heterocycles.

## 1. Introduction

Peptidomimetics, which are considered an essential element in medicinal chemistry, allow for the modulation of protein–protein interactions (PPIs), receptor–ligand interactions, and enzyme inhibition. Numerous research articles have been published to show a diversity of peptidomimetic applications in different therapeutic areas; these applications have included anticancer [1,2], immunomodulating [3], and antiobesity [4] agents, among others. Currently, several peptidomimetics are undergoing clinical trials, and some have been approved as therapeutics [5,6,7]. Peptidomimetics overcome the limitations of small molecules and peptides and provide the desired pharmacophores of the molecules. Over the last decades, new peptidomimetic scaffolds have been presented with the intention of increasing the stability and binding affinity of molecules through conformational regulations. Privileged scaffolds include azoles; piperazine and pyrazine derivatives; quinolines; diazepine; bi- and tricyclic scaffolds; and more. Although peptidomimetics do not possess a natural peptide backbone structure, they can interact with the target protein by arranging essential functional groups, pharmacophores, in a required pattern that is complementary to a binding pocket in a protein [8]. Since a majority of biological processes are mediated by protein secondary structure motifs at the active sites, like helices, turns, and sheets, protein secondary structure mimetics are one of the most attractive design strategies in the peptidomimetic development area. The design of these molecules can be based on both peptide and nonpeptide (small molecule) fragments. This review focuses on the solid-phase synthesis strategies for protein secondary structure mimetics and covers some miscellaneous examples in the last section.

The development of convenient and rapid synthesis methodologies has been an important step in peptidomimetic development. Solid-phase synthesis and combinatorial chemistry techniques have proven to be particularly advantageous instruments for peptidomimetic synthesis. The synthesis of peptidomimetics usually proceeds via solution-phase and solid-phase synthesis methodologies, or their combination. Particularly, solid-phase synthesis provides rapid and convenient access to the large arrays of peptidomimetic analogs. Based on the solid-phase peptide synthesis (SPPS) strategy [9], solid-phase synthesis opens new horizons for enhancing the variety of structural features to be found in peptidomimetics, allowing facile isolation of intermediates by simple filtration, while the recent development of automated methods offers multiple synthesis technologies [10]. Rink amide resin, TentaGel resins, 2-chlorotrityl chloride (2-CTC resin), and Wang resin are the most common solid supports used for synthesis, and classical 9-fluorenylmethoxycarbonyl (Fmoc) or tert-butyloxycarbonyl (Boc) SPPS chemistry is usually applied.

## 2. Solid-Phase Synthesis of α-Helix Mimetics

α-Helices are the most common secondary structural motif observed at PPI active sites (>40% of proteins are helical). Structural features, design concepts, and PPI regulatory mechanisms have been previously covered [11,12,13,14,15]. Privileged scaffolds for α-helix mimetics include terphenyls, tris(2-pyridylmethyl)amines, α,α-dialkyl amino acids, and cross-linked interfacial peptides, among others [16].

**Scheme 1:**
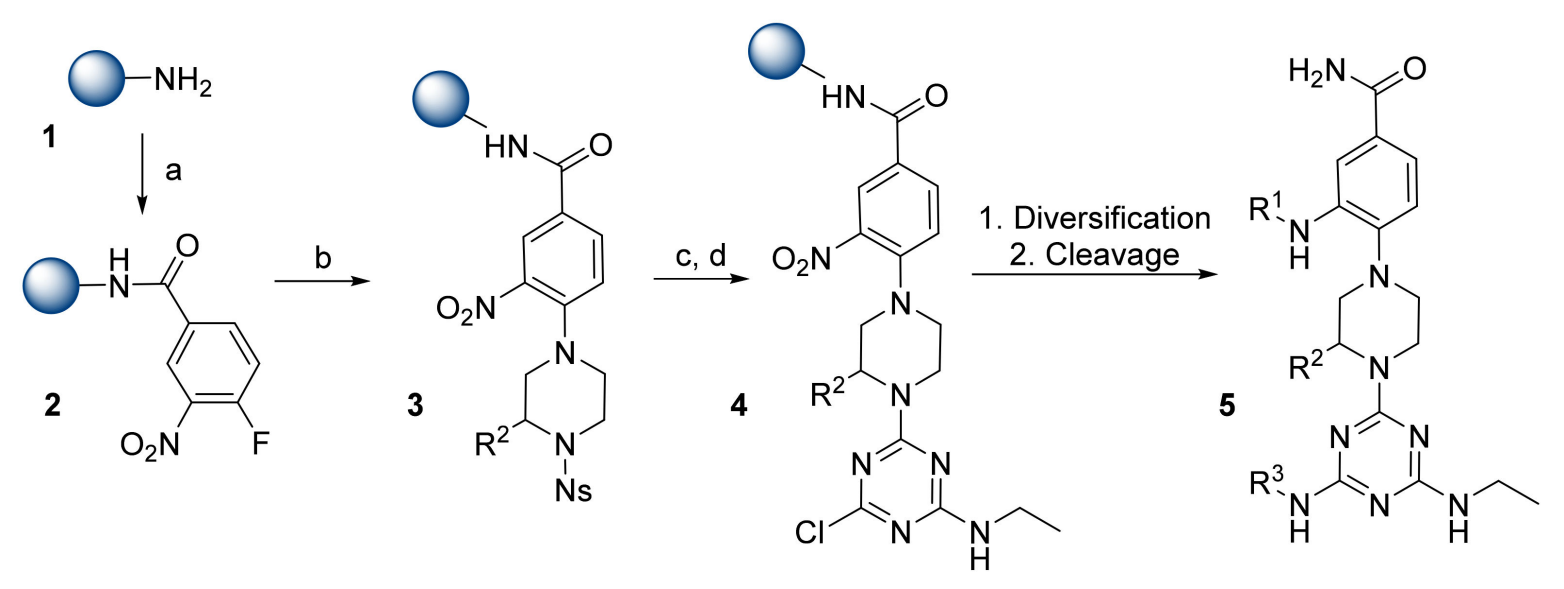
Reagents and conditions: (a) 4-Fluoro-3-nitrobenzoic acid, 1-[bis(dimethylamino)methylene]-1*H*-1,2,3-triazolo[4,5-*b*]pyridinium 3-oxid hexafluorophosphate (HATU)/ *N,N*-diisopropylethylamine (DIPEA), dimethylformamide (DMF), 24 h; (b) Ns-protected piperazines, DIPEA, DMF, 95 °C, 12 h; (c) 2-mercaptoethanol, 1,8-diazabicyclo[5.4.0]undec-7-ene (DBU), DMF, 3 h; (d) 2-aminoethyl-4,6-dichloro-[1,3,5]triazine, DIPEA, tetrahydrofuran (THF), 60 °C, 3 h.

The research team of Lim et al. introduced α-helix mimetics based on the phenyl-piperazine-triazine core that can effectively mimic the key side-chain residues with three functional groups: R^1^, R^2^, and R^3^ [17]. The synthesis starts from the loading of 4-fluoro-3-nitrobenzoic acid on the Rink amide 4-methylbenzhydrylamine hydrochloride (MBHA) resin **1** (Scheme 1). Nosyl (Ns)-protected piperazines were introduced next, followed by coupling with 2-aminoethyl-4,6-dichloro-[1,3,5]triazine. Further diversification and cleavage from the resin resulted in phenyl-piperazine-triazine derivatives **5** in high purities (>85%).

**Scheme 2:**
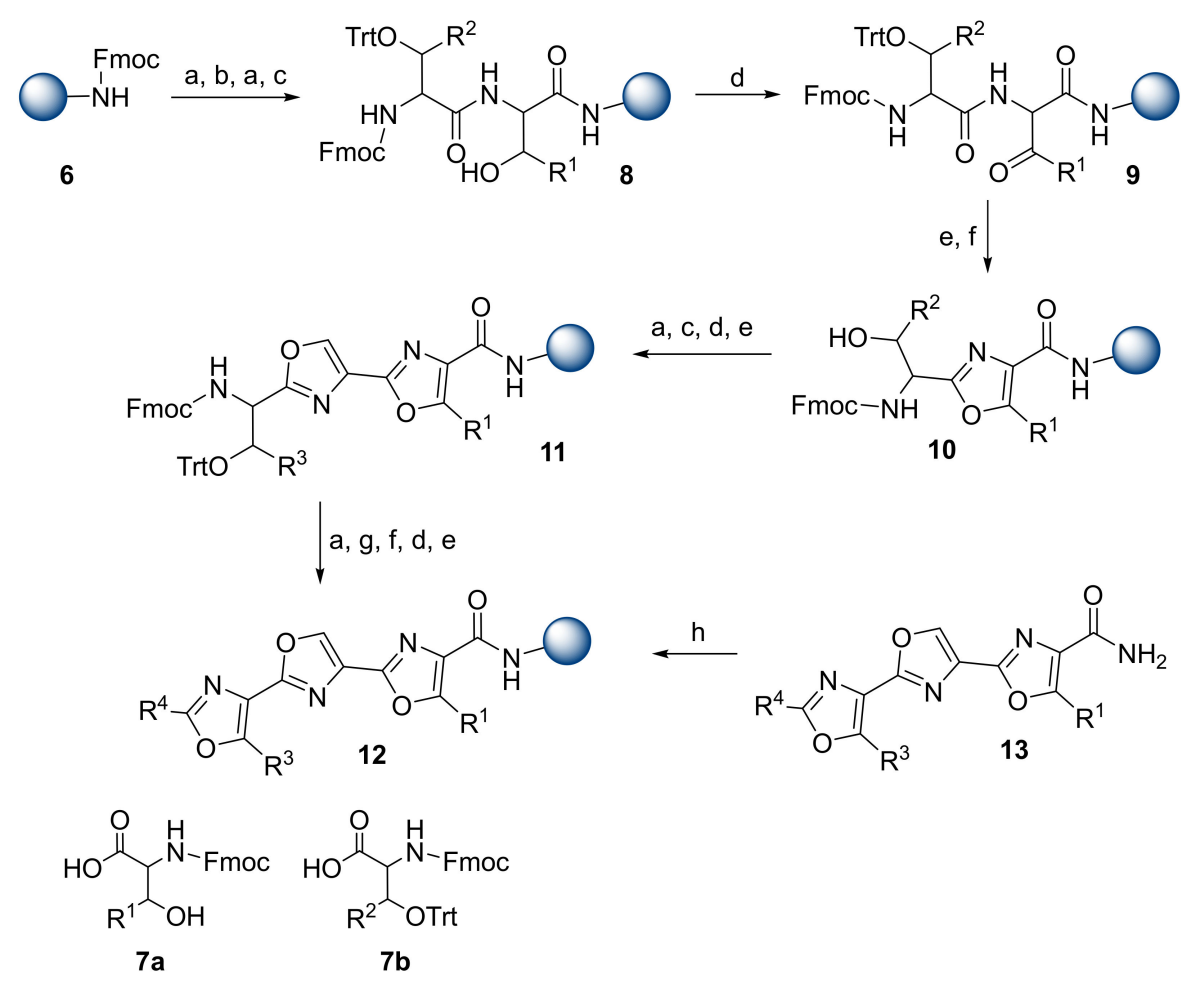
Reagents and conditions: (a) Piperidine/DMF (1/4); (b) **7a**, HOBt, DIC, 1-methyl-2-pyrrolidinone (NMP); (c) **7b**, HOBt, DIC, NMP; (d) DMP, DCM; (e) PPh_3_, I_2_, DIPEA, DCM; (f) TFA/ Triisopropylsilane (TIPS)/DCM (1/5/94); (g) carboxylic acid, HOBt, DIC, NMP or acetic anhydride, DIPEA, NMP; (h) TFA/TIPS/H_2_O (95/2.5/2.5).

Gomes et al. were interested in the synthesis of teroxazole-based α-helix mimetics [18]. The synthesis of teroxazoles started with the preparation of building blocks during the solution-phase. The solid-phase synthesis was performed on a Rink amide MBHA resin **6** using Fmoc/trityl (Trt) chemistry (Scheme 2). The orthogonally protected β-hydroxy-α-amino acids **7** were obtained by aldol addition of protected glycine derivatives with various aldehydes. Amide coupling proceeded in the presence of 1-hydroxybenzotriazole (HOBt) and *N*,*N*′-diisopropylcarbodiimide (DIC). After the conversion of the hydroxy-intermediate to the β-keto amides via Dess–Martin periodinane (DMP), Robinson–Gabriel cyclodehydration was performed using PPh_3_/I_2_ to yield 1,3-oxazoles. Cleavage of the trityl ether was achieved with 1% trifluoroacetic acid (TFA) in dichloromethane (DCM) in the presence of a scavenger. Repetitive coupling and cyclization reactions with the following cleavage from the resin provided target teroxazole compounds **13** in 19–24% yields.

**Scheme 3:**
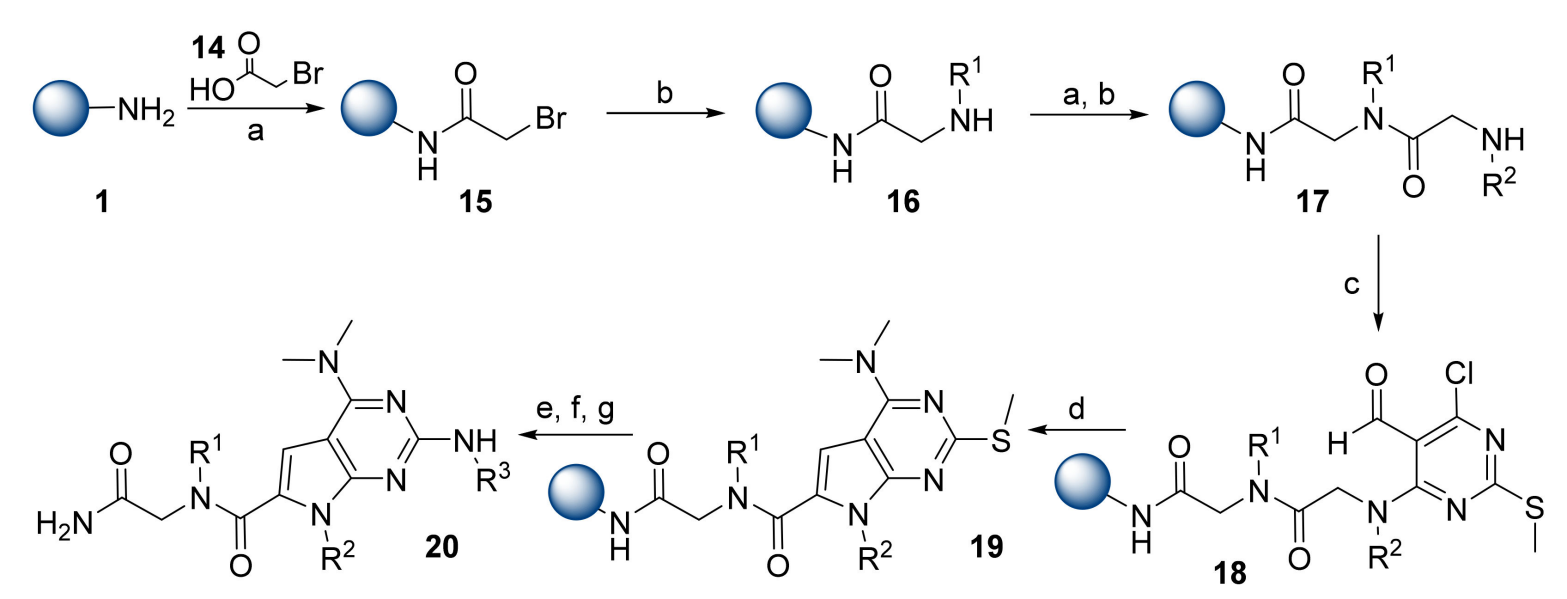
Reagents and conditions: (a) **14**, DIC, DMF; (b) R^1^NH_2_ or R^2^NH_2_, DMF; (c) 4,6-dichloro-2-(methylthio)pyrimidine-5-carbaldehyde, DIPEA, DMF; (d) DBU, DMF, MeOH, 90 °C; (e) 3-chloroperbenzoic acid (mCPBA), NaHCO_3_, DCM; (f) R^3^NH_2_, DIPEA, NMP, 170 °C; (g) TFA/TIPS/H_2_O (95/2.5/2.5), 2 h.

Lim et al. introduced novel pyrrolopyrimidine-based α-helix mimetics [19], and the design of the molecular scaffold was based on Hamilton’s terephthalamide structure [20]. Peptoid synthesis chemistry was used for the solid-phase synthesis of compounds that were assembled on Rink amide MBHA resin **1** (Scheme 3). Pyrrolopyrimidines **19** were formed by the reaction of dipeptoids **17** with 4,6-dichloro-2-(methylthio)pyrimidine-5-carbaldehyde, followed by DBU treatment. Compound **19** then underwent oxidation of the thioether group, amine replacement, and cleavage from the resin that provided pyrrolopyrimidine derivatives **20**. Using a diverse number of primary amines, the research group synthesized a 900-membered library with overall yields from 26% to 40%. The quality-control liquid chromatography–mass spectrometry (LC/MS) check showed the final compounds to have a high average purity (83%).

**Scheme 4:**
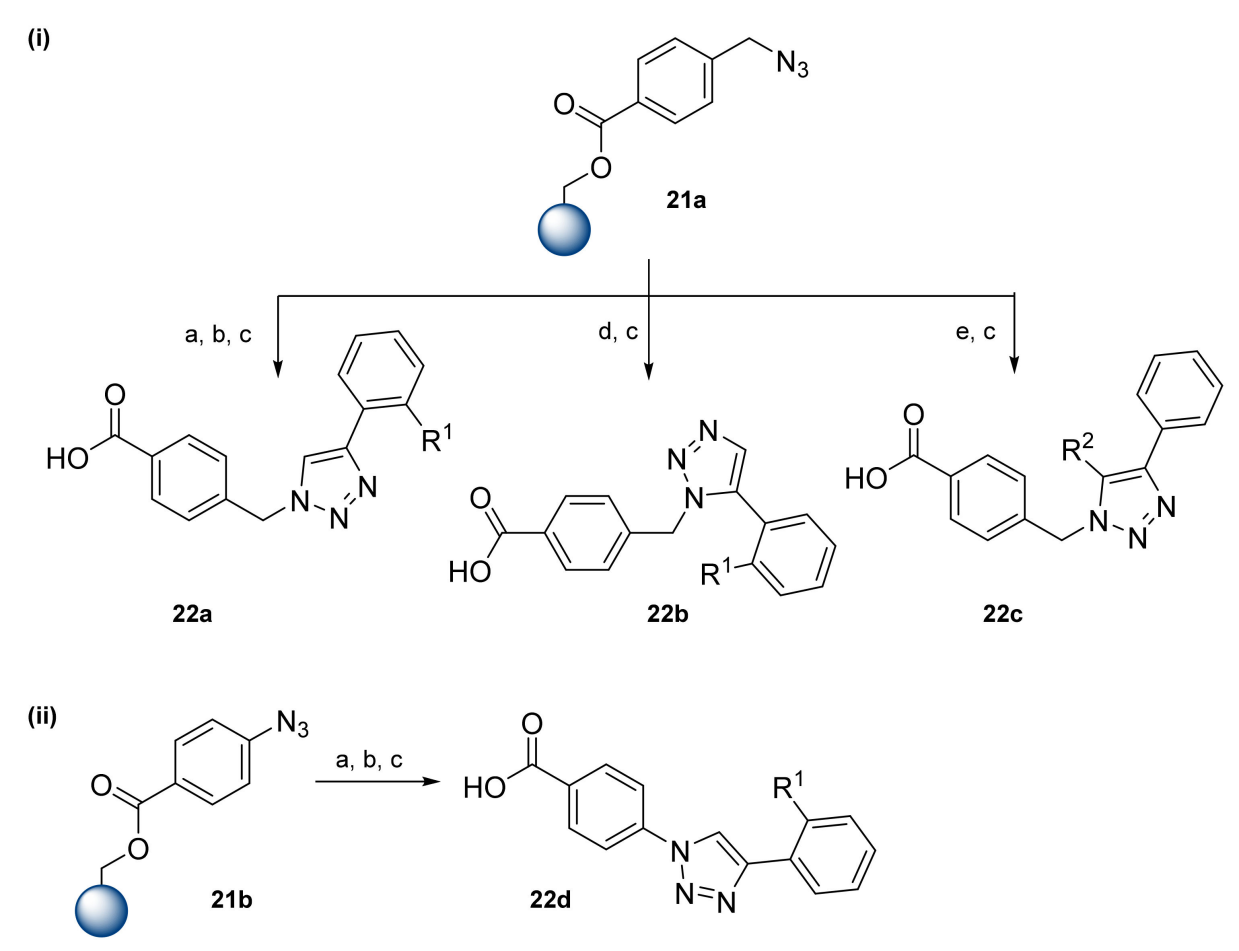
Reagents and conditions: (a) Terminal alkyne, CuSO_4_·_5_H_2_O, L-ascorbic acid, DMF, 24 h; (b) EDTA, DMF, 10 min; (c) TFA/DCM, 2 × 10 min; (d) terminal alkyne, Cp*RuCl(PPh_3_)_2_, DMF, 50 °C, 24 h; (e) internal alkynes, Cp·RuCl(PPh_3_)_2_, DMF, 50 °C, 24 h.

Wrobel et al. presented triazole-based α-helix mimetics prepared by azide–alkyne cycloaddition (Scheme 4) [21]. The parallel solid-phase synthesis starts from the preparation of azide derivatives and is followed by loading onto the Wang resin via an esterification reaction providing resins **21**. Resins **21** were subjected to an azide–alkyne cycloaddition using alkynes in the presence of copper (II) or a ruthenium catalyst. First, azide-bond resin **21a** was treated with terminal alkynes in the presence of CuSO_4_·5H_2_O and L-ascorbic acid (Scheme 4i, route (a). After the removal of copper salts with ethylenediaminetetraacetic acid (EDTA) and cleavage from the resin with TFA, the resulting 1,4-disubstituted triazoles **22a** were obtained in 88–99% yields. The application of a ruthenium(II) catalyst resulted in 1,5-disubstituted triazoles **22b** in 43–96% yields (Scheme 4i, route (d). Terminal alkynes were replaced with internal alkynes, and a ruthenium (II) catalyzed cycloaddition afforded 1,4,5-trisubstituted triazole derivatives **22c** in 78–98% yields (Scheme 4i, route e). Resin **21b** with an aromatic azide was treated with a copper (II) catalyst in the same manner; however, the resulting triazole products **22d** were obtained in lower yields, 21–63% (Scheme 4ii). This can be explained by the steric effect of the aromatic ring. Overall, Wrobel et al. synthesized compounds with two fixed rings, benzene and triazole, while the third ring contained various substituents, including both hydrophobic and hydrophilic functional groups, including bulky groups like naphthalene or phenanthrene.

## 3. Solid-Phase Synthesis of Turn Mimetics

Perhaps of the most importance after α-helices, organized turn motifs are among the most well-studied secondary structural features of proteins [22,23]. Generally, turn lengths can vary from three to six amino acids, and their irregular secondary structural elements differ from the dihedral angles (φ and ψ) that constitute their backbones [24]. The privileged structures for the turn mimetics include benzodiazepines, monosaccharides [25], and cyclopentapeptides, among others [16,26]. 

**Scheme 5:**
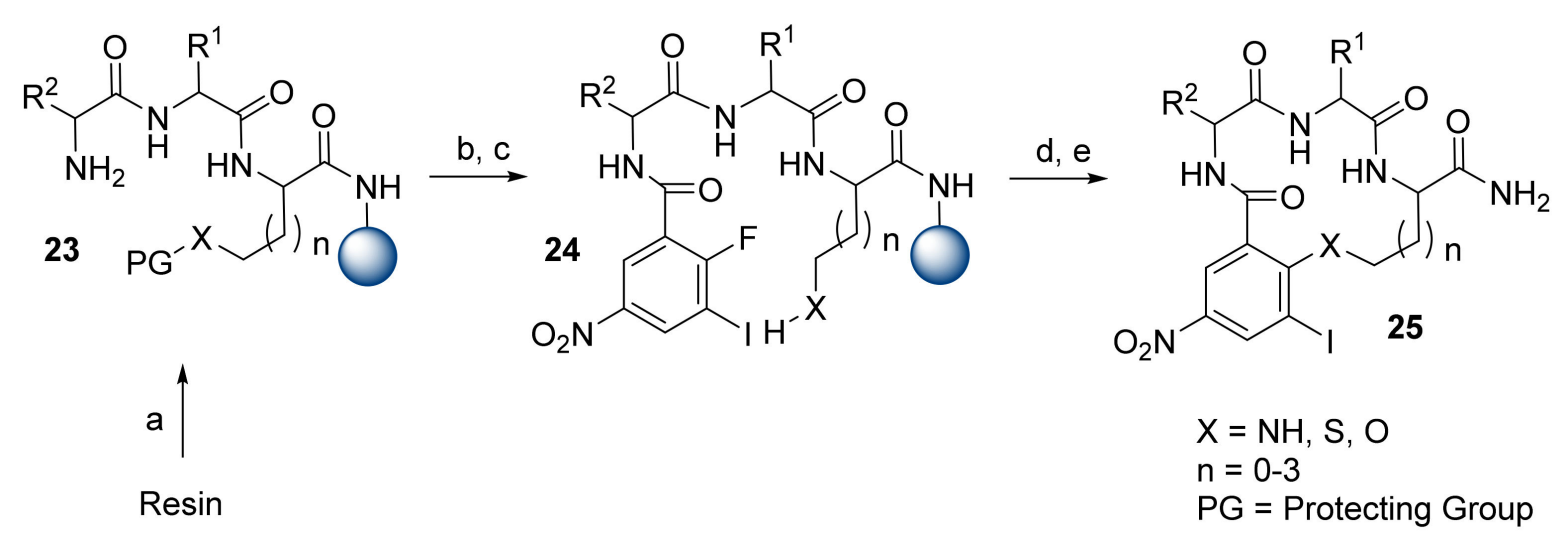
Reagents and conditions: (a) DIC/ 4-methylmorpholine (NMM) Fmoc SPPS; (b) 2-fluoro-3-iodo-5-nitrobenzoyl chloride, DIPEA, DCM, 2 h; (c) TFA/TIPS/DCM (1/5/94), 5 × 5 min; (d) 0.1 M K_2_CO_3_, DMF, 36 h; (e) TFA/TIPS/DCM (1/5/94), 4 h.

The research team of Burgess worked continuously on the development of β-turn mimetic scaffolds. Although the β-turn mimetic scaffolds presented by Burgess do not contain heterocycles, it is still worth mentioning the β-turn mimetic synthesis approaches developed by the team. Their first reports were based on a solid-phase S_N_Ar macrocyclization approach [27,28,29]. The synthesis was performed on Rink Amide MBHA and TentaGel S RAM resins (Scheme 5). It was observed that the formation of the *N*- and *S*-macrocycles occurred in higher yields on a Rink Amide MBHA resin, while the formation of the *O*-macrocycles occurred in higher yields on the TentaGel S RAM resin. To obtain *N*-macrocycles, Gln was loaded at the first position, degraded via an on-resin Hofmann degradation (PhI(O_2_CCF_3_)_2_, H_2_O, DMF, 16 h), and then protected with 4-methyltrityl (Mtt) chloride (DCM, DIPEA, 2 h). The Ser derivatives were formed using the Trt-protected amino acids, while, to form the sulfur heterocycles, Cys was used at the first position with the 4-methoxypheny(diphenyl)methyl (Mmt) protection. Tripeptide synthesis proceeded based on standard Fmoc SPPS protocols, followed by the introduction of the 2-fluoro-3-iodo-5-nitrobenzoyl group. The protecting group (Mtt, Trt, or Mmt) was removed and the key reaction, S_N_Ar macrocyclization, was performed using 0.1 M K_2_CO_3_ in DMF at room temperature (RT) for 36 h. After cleavage, compounds **25** were obtained in 49–73% yields and 89–98% purities. Additionally, on-resin functionalization of the aryl iodides proceeded via Sonogashira and Suzuki couplings, resulting in 68 and 45% yields, respectively [28].

**Figure 1:**
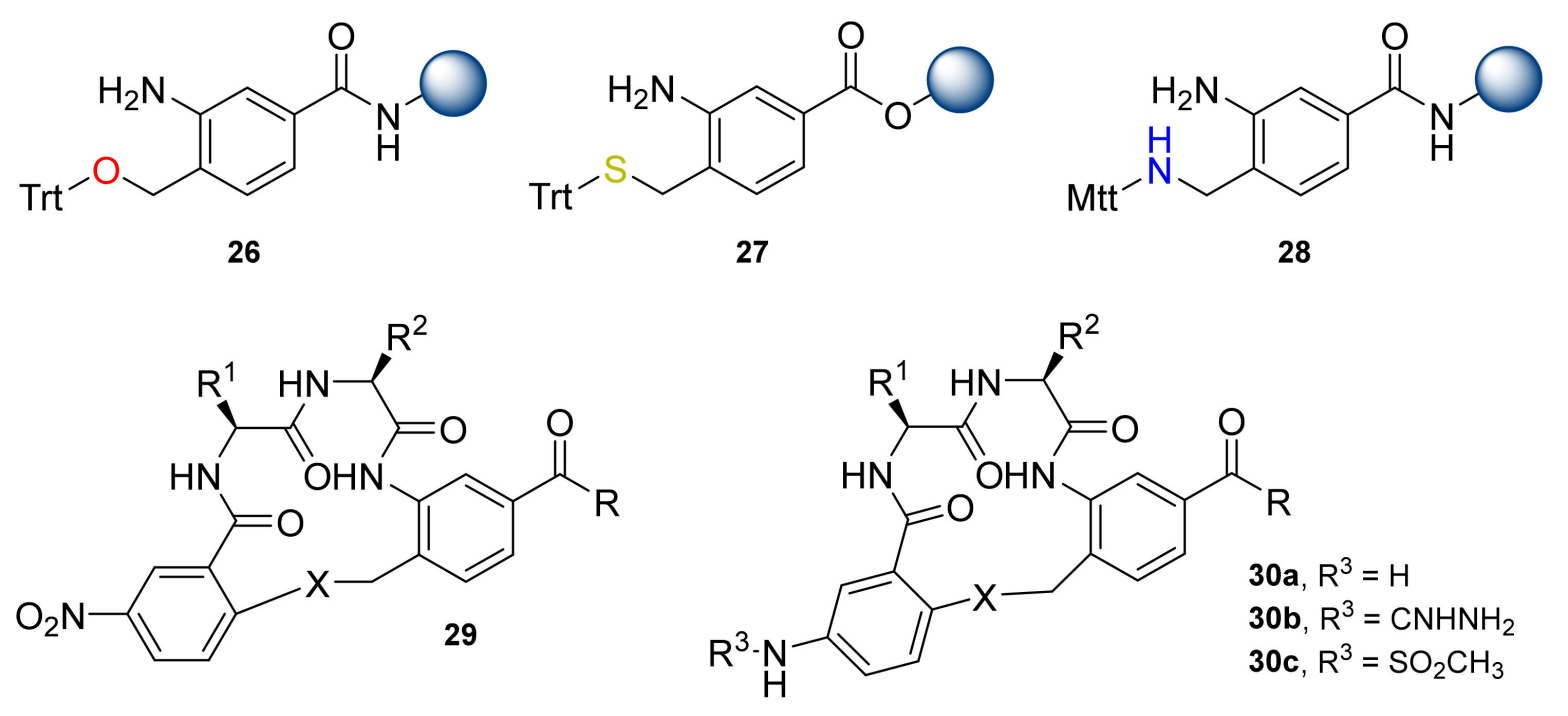
C^10^ β-turn mimetics and resins for their synthesis.

Second generation β-turn mimetic (C^10^ β-turn mimetic) templates were presented later. *O*-, *S*-, *N*-containing solid-supported templates **26–28** were prepared via various modifications of 4-(bromomethyl)-3-nitrobenzoic acid (Figure 1) [30]. Several resins were tested, and the Rink amide resin was best for coupling reactions for *O*-, *N*-containing solid-supported templates, whereas the Wang resin was used for the synthesis of the *S*-containing templates, due to the unsuitable Trt deprotection conditions from the sulfur. Compounds **26**–**28** underwent consecutive couplings following the addition of the 2-fluoro-5-nitro benzoyl group and further cyclization. Products **29** were obtained in moderate to high yields. To improve the solubility and bioavailability of these compounds, the NO_2_ moiety was replaced with a primary amine, guanidine, and methyl sulfoxide groups to give the corresponding products **30a**, **30b**, and **30c**, respectively [31]. Inspired by the cyclic mimetic structure reported by Burgess, Li et al. presented a one-pot solid-phase synthesis of the β-turn cyclic mimetics using “volatilizable” aminoalkyl silica gel as a polymer support [32]. The chain elongation proceeded on silica gel, and the macrocyclization was performed after cleavage using an ion exchange resin consisting of equivalent amounts of the strong anion (OH⁻) and strong cation (H⁺) in 10% aqueous acetonitrile (ACN). Cyclized compounds were obtained in 70–92% yields and purities ≥ 76%.

**Scheme 6:**
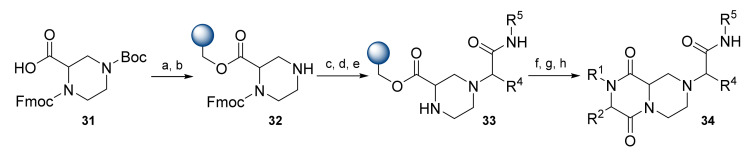
Reagents and conditions: (a) Hydroxymethyl polystyrene resin, Ph_3_P, diethyl azodicarboxylate, THF; (b) TFA/DCM, 1 h; (c) OHCCO_2_H·H_2_O, R^4^B(OH)_2_, DCM; (d) DIC, R^5^NH_2_, DCM; (e) 25% piperidine in DMF; (f) Boc-AA-OH, (benzotriazol-1-yloxy)tripyrrolidinophosphonium hexafluorophosphate (PyBOP), DMF; (g) 2% TFA in DCM, 1 h; (h) 2 M AcOH, *i*BuOH, 50 °C, 24 h.

Golebowski et al. presented a series of diketopiperazine-based β-turn mimetics. The first report focused on diketopiperazine formation via a Petasis reaction (Scheme 6) [33]. The synthesis started with attachment of the piperazine-2-carboxylic acid **31** to the hydroxymethyl polystyrene resin. The Boc group was removed, and the liberated amine of **32** underwent Petasis reaction with glyoxylic acid monohydrate and various boronic acids. The resulting intermediate was coupled with amines, and then with *N*-Boc-protected α-amino acids (AA). Deprotection and cyclative cleavage then afforded final compounds **34** in 56–96% overall yields and purities of 70–88%.

**Scheme 7:**
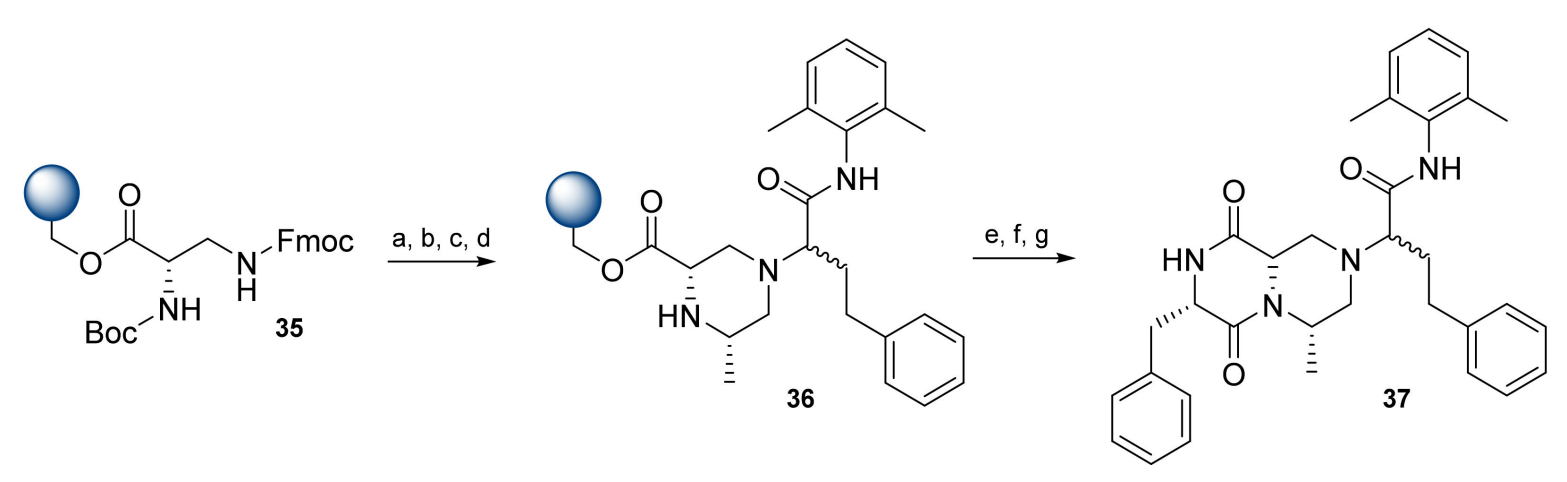
Reagents and conditions: (a) 25% piperidine, DMF, 30 min; (b) hydrocinnamaldehyde, 2,6-dimethylphenyl isocyanide, *(R)*-( + )-2-bromopropionic acid, MeOH/CHCl_3_ (1/4), RT, 2 × 2 h; (c) 25% TFA/DCM; (d) 10% DIPEA, DCM, 18 h; (e) Boc-Phe-OH, NMM, isobutyl chloroformate, THF, DMF; (f) 25% TFA/DCM; (g) 2 M AcOH, IPA, 50 °C, 18 h.

Derivatives of compound **34** lack an *i* + *3* side-chain substituent, thus a new approach with the incorporation of α-*N*-Boc-β-*N*-Fmoc-L-diaminopropionic acid was introduced (Scheme 7). A Ugi reaction of **35** with hydrocinnamaldehyde, 2,6-dimethylphenyl isocyanide, and (*R*)-( + )-2-bromopropionic acid afforded an intermediate that was then cyclized to provide resin-bound piperazine **36**. Cyclative release via AcOH in isopropyl alcohol (IPA) afforded compound **37** in a 20% overall yield (average 82% yield per step) and purity of 85%. Due to the low diastereoselectivity of the Ugi reaction, the crude product **37** was isolated as a 1:1.5 mixture of diastereoisomers [34]. Based on this approach, solid-phase synthesis of the diketopiperazine-based β-turn mimetic library was accomplished, affording derivatives of **37** with 50–90% purity [35].

**Scheme 8:**
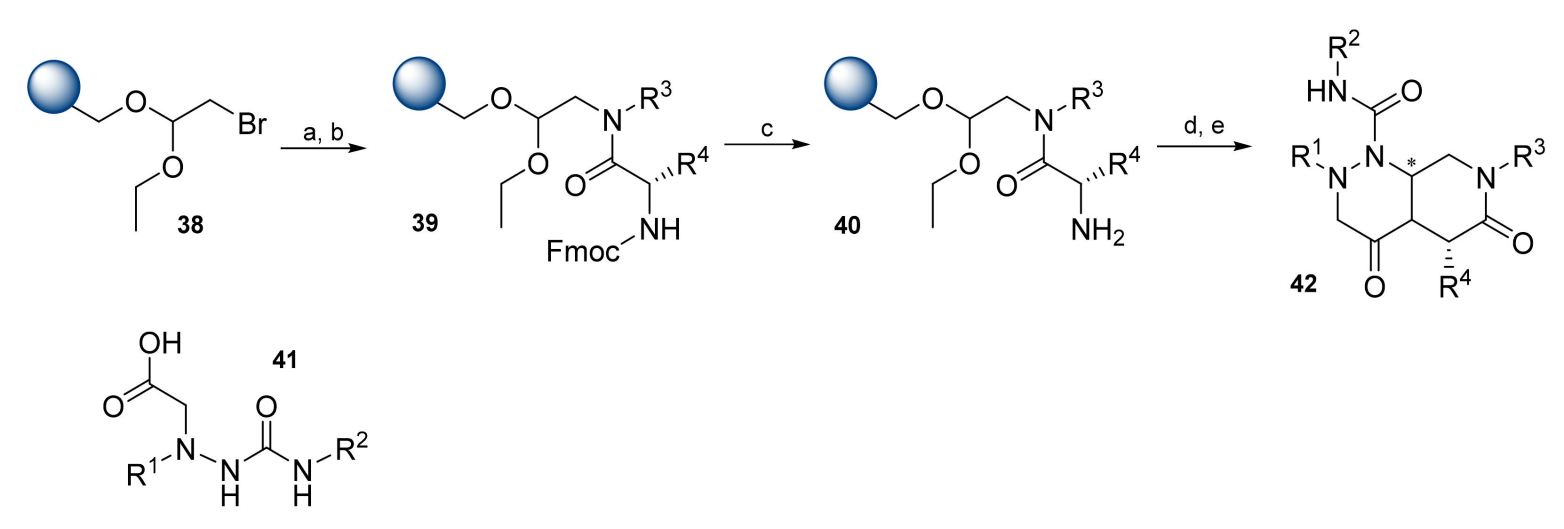
Reagents and conditions: (a) R^3^NH_2_, dimethyl sulfoxide (DMSO), 60 °C, 12 h; (b) Fmoc-AA-OH, HCTU/DIPEA, DMF, 4 h; (c) 20% piperidine, DMF, RT, 10 min; (d) **41**, DIC/HOBt, DMF, 3 h; (e) HCOOH, 18 h.

Kim et al. developed a solid-phase synthesis methodology for the synthesis of β-turn mimetic-based leucyl-tRNA synthetase (LRS)-RagD PPI stabilizers. The solid-phase synthesis was performed on bromoacetal resin **38** (Scheme 8) [36]. Various amines were loaded onto the resin via an S_N_2 reaction, and then coupled with natural and unnatural Fmoc amino acids using *O*-(1*H*-6-chlorobenzotriazole-1-*yl*)-1,1,3,3-tetramethyluronium hexafluorophosphate (HCTU)/DIPEA, followed by coupling with compound **41**. The key step was the formation and release of bicycle **42** under neat treatment with formic acid. A diverse 162-membered library based on **42** was obtained in high purities.

**Figure 2:**
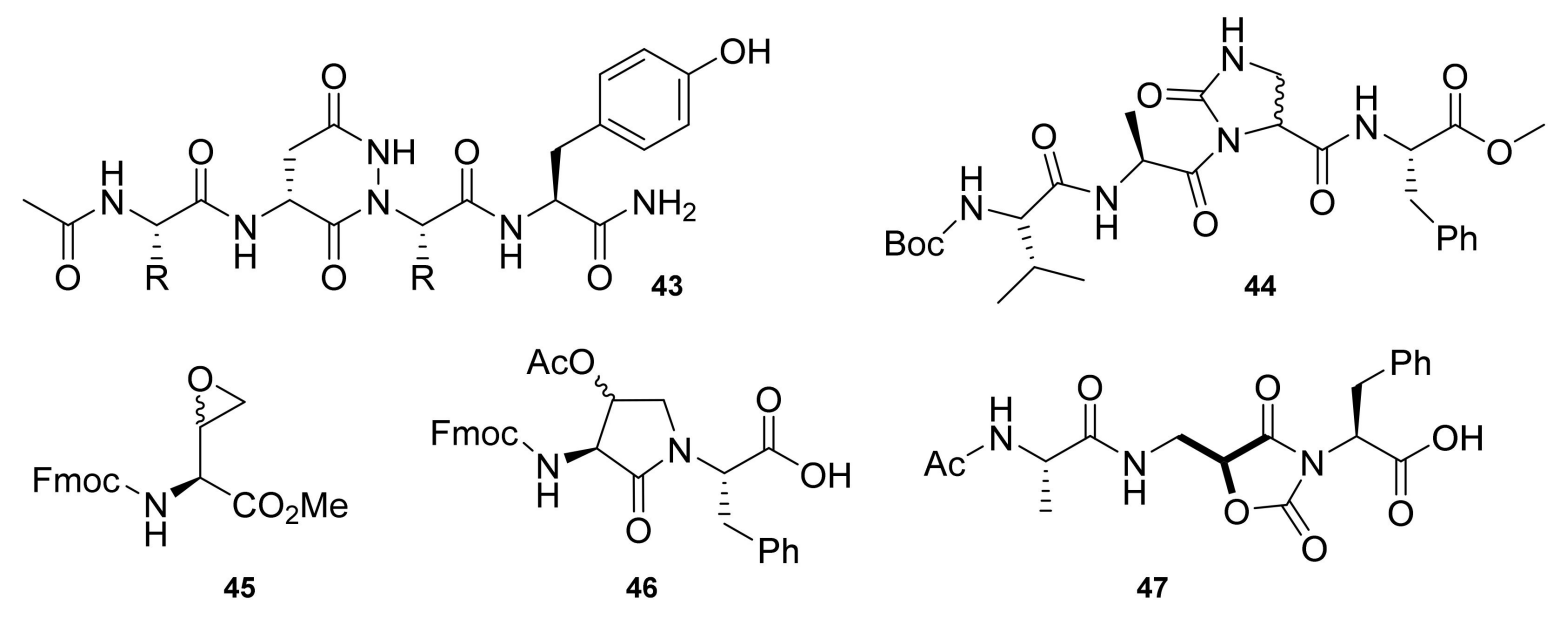
Mimetics based on peptide modifications.

Kang et al. prepared a solid-phase synthesis of tetrahydropyridazine-3,6-dione (Tpd)-constrained peptidomimetics from α-hydrazino acids (**43**, Figure 2) [37]. Solid-phase synthesis on a Rink Amide MBHA resin via Fmoc SPPS afforded Tpd peptidomimetics **43** in 3–42% yields. De Marco et al. presented another example of a mimetic, by developing (*S*)- and (*R*)-imidazolidin-2-one-4carboxylates (Imi-peptides) on a solid-phase from (*S*)- or (*R*)-α,β-diaminopropionic acid residues (**44**, Figure 2) [38]. A two-step procedure for the synthesis of peptidomimetics containing cyclic threonine (cThr) was presented by Sicherl et al. [39]. Their synthetic strategy was based on the addition of an amino group to epoxide. An epoxide opening reaction was accomplished by the addition of Ca(OTf)_2_ to a mixture of compound **45** and amino acid under microwave (MW) irradiation. Subsequently, ring closure was completed by heating with toluene for 12 h, resulting in a dipeptide building block **46** with a cThr moiety (78% yield). The peptidomimetic molecule on a solid-phase was constructed on a 4-(2′,4′-dimethoxyphenyl-Fmoc-aminomethyl)-phenoxyacetamido-aminomethyl (Rink amide AM-PS) resin using HATU/ 1-hydroxy-7-azabenzotriazole (HOAt) chemistry. A final compound with a cThr moiety was obtained with a 37% overall yield. Greco et al. presented pseudo β-turn peptidomimetics based on the β^2^- and β^2,2^-homo-Freidinger lactam analogs [40]. Lactam analogs were prepared via single-step cyclization of α-hydroxy-β^2^-amino acids or α-substituted-α-hydroxy-β^2,2^-amino acids present in the peptide chain to 5-aminomethyloxazolidine-2,4-dione rings (Amo). Cyclization of the compounds with *iso*Ser (**47**, Figure 2) was achieved in either a solution- or solid-phase using *N*,*N*′-disuccinimidyl carbonate (1.1 eq. for solution-phase synthesis, 3 eq. for SPPS) and DIPEA (a catalytic amount for solution phase synthesis, 1 eq. for SPPS). Both synthetic methods provided Amo-peptides in high yields (≥ 80%).

**Scheme 9:**
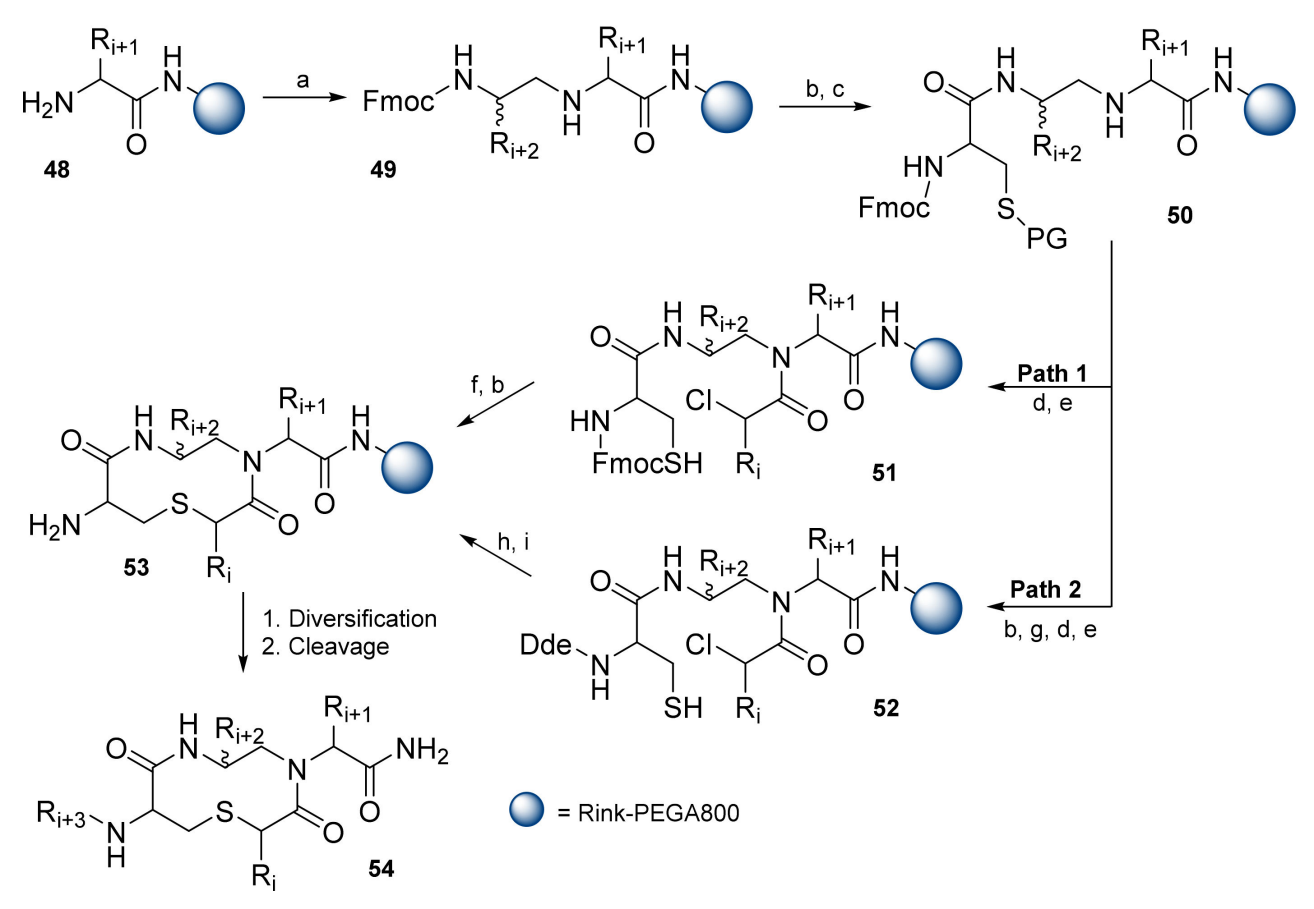
Reagents and conditions: (a) Fmoc amino aldehyde, NaBH_3_CN, AcOH, DMF; (b) 20% piperidine in DMF; (c) Fmoc-Cys(*t*Bu)-OH, *O*-(benzotriazol-1-*yl*)-*N,N,N′,N′*-tetramethyluronium tetrafluoroborate (TBTU)/NEM, DMF; (d) (ClCH_2_CO)_2_O, NEM, DCM or PhCHClCOCl, DIPEA, DCM; (e) Bu_3_P, H_2_O, THF; (f) NEM, DMF, 55–60 °C, 7 h; (g) 2-acetyldimedone, DMF; (h) DBU, DMF; (i) 3% hydrazine/DMF.

Bondebjerg et al. reported on the solid-phase synthesis of a thioether cyclized peptidomimetic scaffold. The synthesis proceeded on poly[acryloyl-bis(aminopropyl)polyethylene glycol] (PEGA) resin with a Rink amide linker (Scheme 9) [41]. The first amino acid was introduced using standard Fmoc coupling conditions resulting in resin **48**. Reductive alkylation using Fmoc amino aldehyde in the presence of NaBH_3_CN resulted in resin **49**. Peptidomimetic compounds were prepared in two ways. During the original Path 1, intermediate **50** underwent chloroacetylation with subsequent removal of the tert-butyl (*t*Bu) group from Cys, resulting in resin **51**. Macrocyclization was achieved by heating at 55–60 °C with *N*-ethylmorpholine (NEM) in DMF for 7 h. Due to interest in attempting to lower the reaction temperature, Path 2 was developed. Reducing the temperature required a stronger base to accomplish cyclization and, consequently, the base labile Fmoc protecting group could not be used. Fmoc was replaced by *N*-(1-(4,4-dimethyl-2,6-dioxocyclohexylidene)ethyl (Dde), and cyclization was achieved by the addition of DBU in DMF without heating. Regarding both intermediates, amine was liberated from the protecting groups, resulting in cyclized resin **53**. The primary amine underwent reductive alkylation to introduce R*_i + 3_*. Release from the resin via TFA/TIPS afforded product **54**. 

## 4. β-Strand, β-Sheet, and β-Hairpin Mimetics

**Scheme 10:**
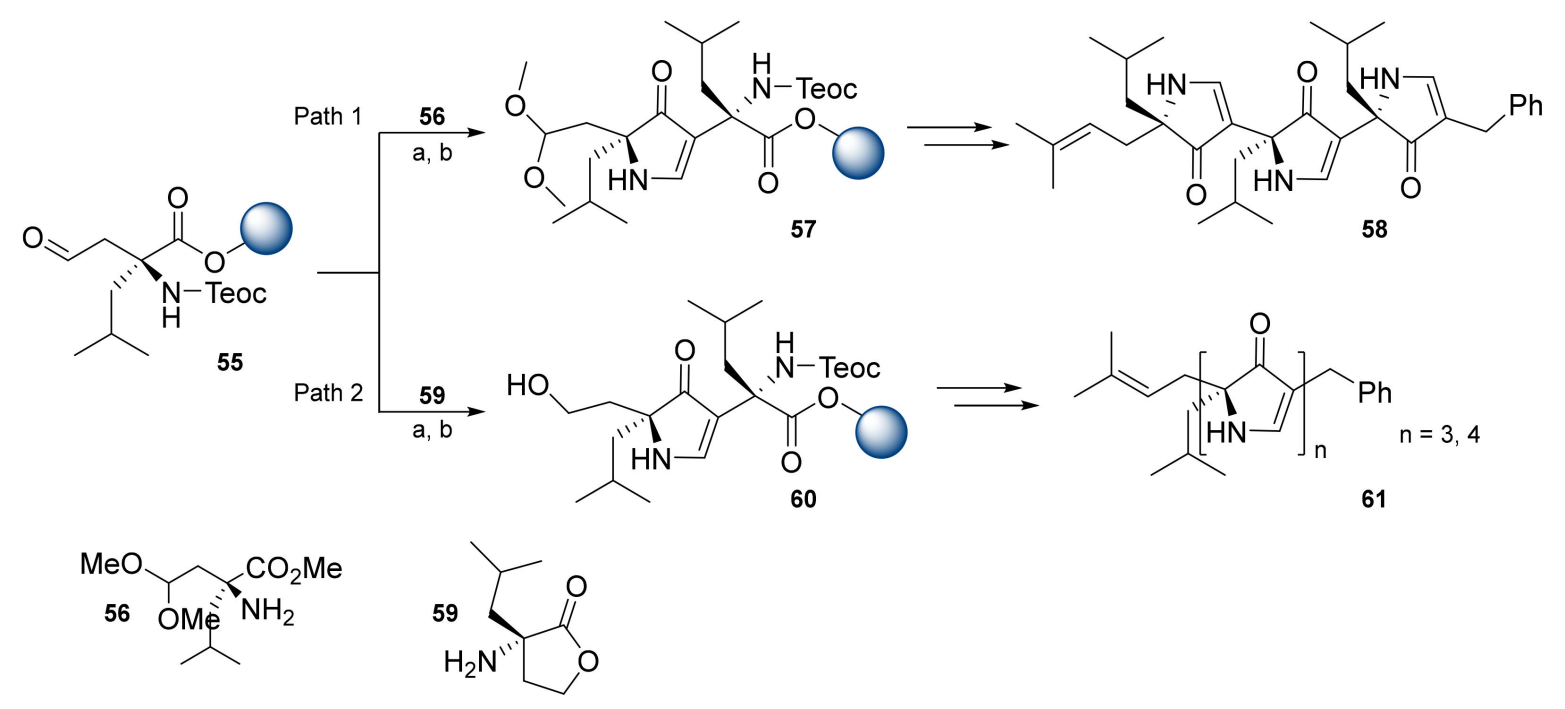
Reagents and conditions: (a) **56** or **59**, (MeO)_3_CH, THF, twice; (b) KHMDS.

The polypyrrolinone structural motif is a privileged scaffold for β-strand and β-sheet mimetics. Smith et al. presented a three-step iterative solid-phase synthesis of polypyrrolinones. Regarding the synthesis of trispyrrolinone, resin-bound intermediate **55** with aldehyde functionality reacted with amino ester **56**, followed by metalloimine cyclization via potassium bis(trimethylsilyl)amide (KHMDS), and yielded resin-bound monopyrrolinone **57** (Scheme 10, Path 1). Treatment of compound **57** with *p*-toluenesulfonic acid at 40 °C provided monopyrrolinone with an aldehyde moiety. The above sequence was repeated and followed by cyclorelease of trispyrrolinone **58** from the resin (8.4% 10-step overall yield) [42]. Unfortunately, this strategy did not work for the synthesis of tetrapyrrolinone: the strong acid hydrolysis conditions along with several oxidation steps failed during the additional iteration. To solve this, researchers proposed an amino lactone-based strategy by the introduction of compound **59** (Scheme 10, Path 2). Iteration of the above sequences (routes a,b) resulted in polypyrrolinones **61** in 13.4% of the 10-step overall yield (*n* = 3) and 2.5% of the 13-step overall yield (*n* = 4) [42].

**Scheme 11:**
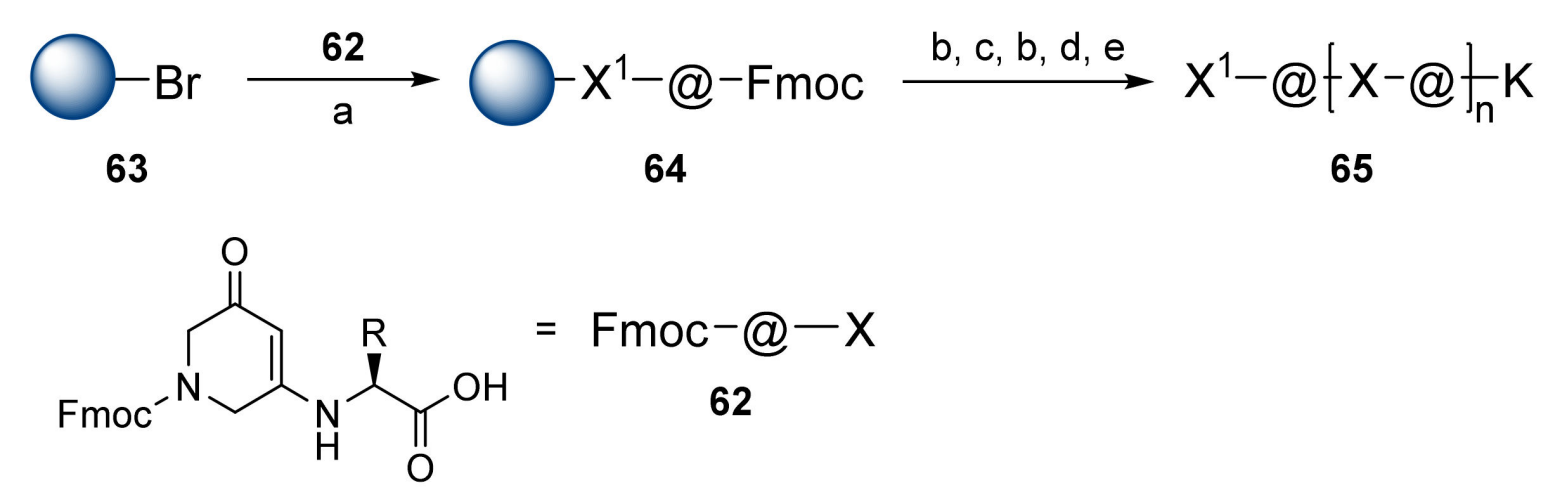
Reagents and conditions: (a) **62**, CsI, DIPEA, DMF; (b) 20% piperidine, DMF; (c) **62**, HATU, DIPEA, DMF; (d) (Boc)_2_Lys, HATU, DIPEA, DMF; (e) TFA/DCM (1/1).

Various amide bond surrogate units have been presented over the years using solid-phase synthesis strategies. Phillips et al. presented β-strand peptidomimetics called @-tides that were composed of alternating amino acids with dihydropyridinones, these @-tide units are seen in **62** (Scheme 11) [43]. The synthesis of di-@-tide building blocks proceeded during the solution-phase via the direct condensation of amino acids with *N*-protected 3,5-piperazinedion [44]. The semiautomated Fmoc SPPS on the brominated Wang resin **63** provided oligomers with up to 13 units in length in isolated yields of 20–80%. Using this methodology, Phillips and co-workers then presented @-tide-stabilized β-hairpins by combining an @-tide β-strand with a β-turn inducer D-Pro-Ala moiety [45].

**Scheme 12:**
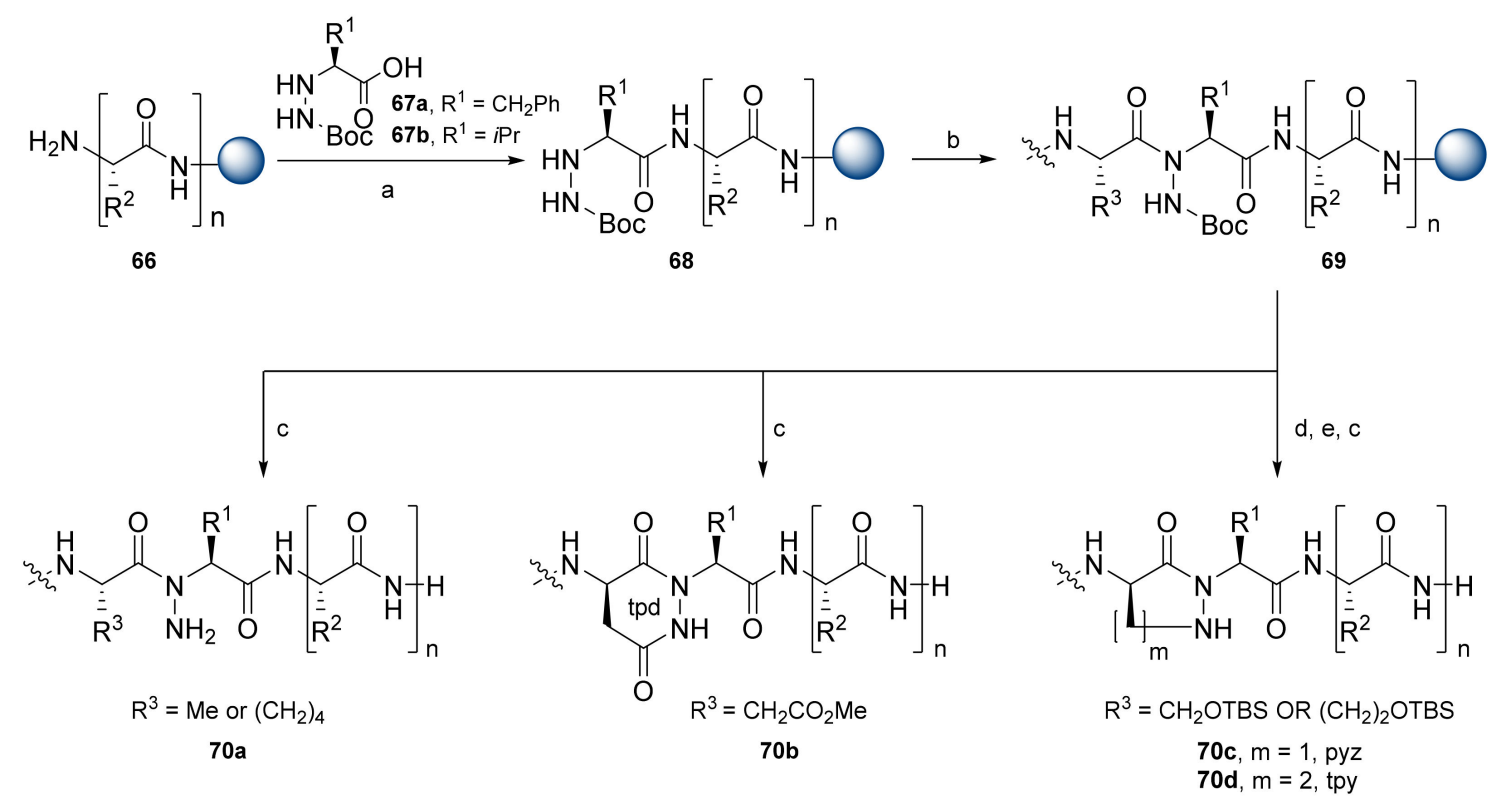
Reagents and conditions: (a) HATU, DIPEA, 50 °C; (b) Fmoc SPPS; (c) TFA: H_2_O (95:5); (d) tetra-*n*-butylammonium fluoride, THF; (e) DIAD, PPh_3_.

The research team of Del Valle presented a new class of β-strand mimetics based on oligomeric tpd units that were previously described (Section 2) [46]. The tpd unit was synthesized by an alternative introduction of α-hydrazino amino acids with D-Asp. Their team later presented β-sheet mimetics with constrained *N*-amino peptides (NAP) [47]. These included β-strand variations with the incorporation of ‘trans-locked’ tpd, (*R*)-tetrahydropyridazinone (tpy), and (*R*)-pyrazolidinone (pyz) scaffolds. These orthotic constraints were readily introduced onto a solid support via lactamization (for tpd) and Mitsunobu reactions (for tpy and pyz) between an *N*-aminated residue and an adjacent D-homoserine or D-serine side chain (Scheme 12). Solid-phase synthesis proceeded on an MBHA resin using an Fmoc SPPS strategy. The coupling of resin-bound peptides **66** with hydrazine amino acids **67** was accomplished with HATU activation yielding intermediate **68**. Hydrazine acids **67** were prepared during the solution-phase from standard amino acids by oxaziridine-mediated electrophilic amination. Coupling to hydrazine amides required prior acid chloride activation: Fmoc-Asp(Me)-Cl for tpd formation was afforded via SOCl_2_, while activation of Fmoc-D-hSer-OH and Fmoc-D-Ser-OH proceeded via bis(trichloromethyl) carbonate and collidine in situ. After peptide elongation with Fmoc chemistry, peptides **69** were cleaved from the resin using a TFA/H_2_O cocktail. Formation of the tpd ring occurred readily during cleavage, yielding compound **70b**. Mitsunobu cyclization using diisopropyl azodicarboxylate (DIAD) and PPh_3_ proceeded in solid support before cleavage. Cleavage from the resin afforded pyz and tpy restrained analogs **70c** and **70d**, respectively.

**Scheme 13:**
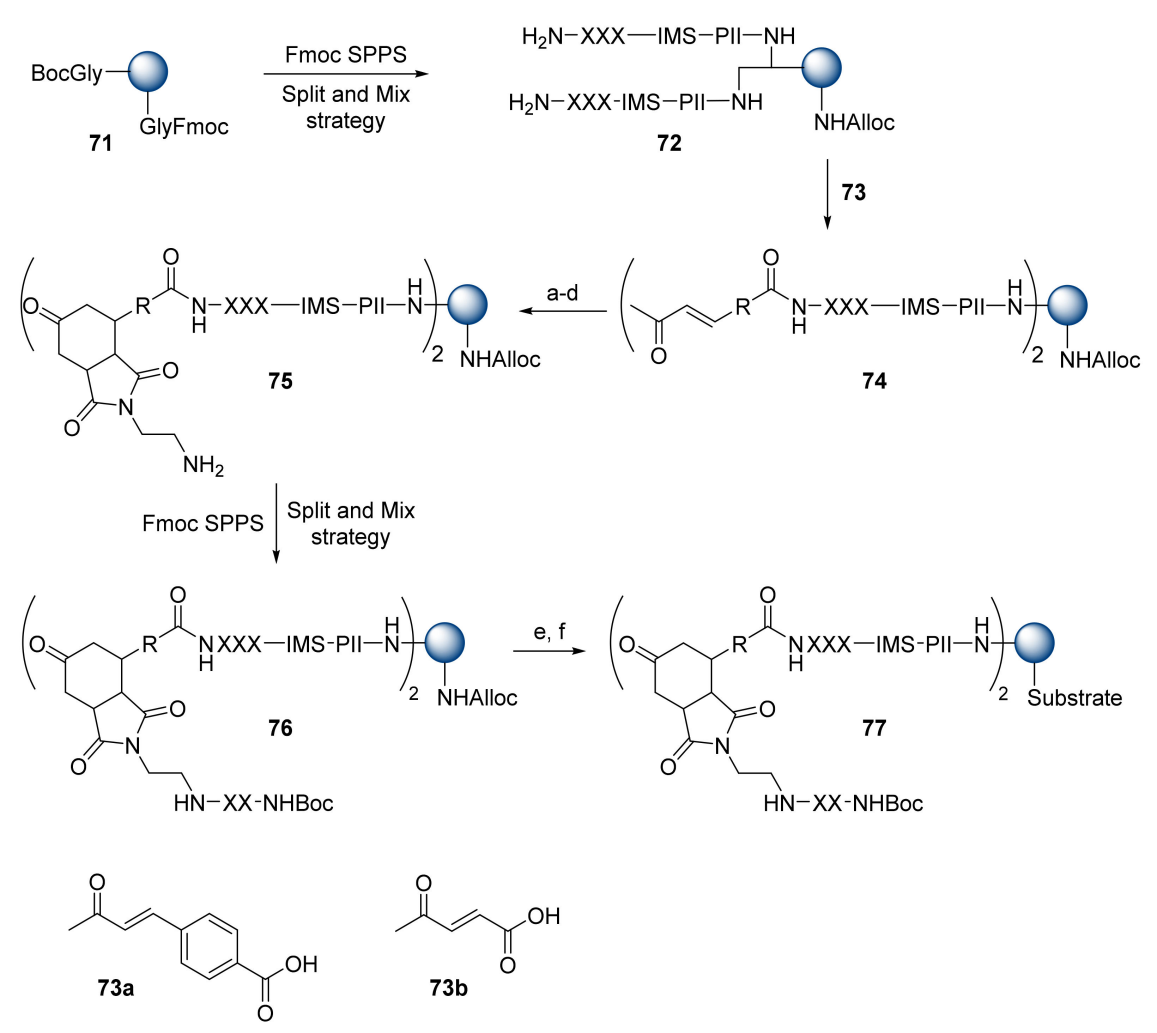
Reagents and conditions: (a) TEA, TBDMSOTf; (b) *N*-(Fmoc-amino)ethylmaleimide; (c) Boc_2_O, NEM; (d) 20% piperidine, DMF; (e) Pd(PPh_3_)_4_, NEM, AcOH; (f) BocY(NO_2_)E(*t*Bu)K(Boc)FR(Pmc)---R(Pmc)GK(Boc)K((Boc)-Abz)G-OH, TBTU/NEM.

Graven et al. reported on a combinatorial split-and-mix synthesis of a “one-bead–two-compounds” library of peptide isosteres, based on Diels–Alder reactions. The library was constructed on PEGA resin, using an orthogonal protecting group strategy (Scheme 13) [48]. First, a mixture of Boc- and Fmoc-protected glycine (1:1) was introduced onto the resin via TBTU chemistry to afford resin **71**. The Fmoc-protected site was converted into Pd-labile *N*-Alloc, to prevent side reactions during further Fmoc SPPS. Occurring at the Boc-protected site, the amino acid sequence was introduced, with the subsequent attachment of a photolabile linker (Pll) and ionization-mass spacer (IMS) sequence for matrix-assisted laser desorption ionization time-of-flight (MALDI-TOF) analysis. The resulting resin was subjected to split-and-mix synthesis in a multiple-column peptide synthesizer, using Fmoc- and Boc-protected amino acids (9:1) with TBTU activation to yield intermediate **72**. Two equal portions of **72** were reacted with **73** to give intermediates **74**. Silyl enolization of the recombined mixture of **74** with tert-butyldimethylsilyl trifluoromethanesulfonate (TBDMSOTf) and triethylamine (TEA) provided activated diene and was followed by a reaction with *N*-(2-((*N*-fluoren-9-ylmethoxycarbonyl)aminoethyl maleimide in toluene overnight at 80–85 °C. Being unstable to Diels–Alder reaction conditions, the Boc-protecting group was reintroduced, and the Fmoc-group removed for further SPPS. Two more amino acids were introduced to intermediate **75** via split-and-mix synthesis methodology giving **76**. To analyze the quality of the synthesized library of **76**, 72 beads were subjected to cleavage using a TFA/scavenger cocktail. MALDI-TOF analysis showed that 73% of the beads had a full sequence assignment, while 69% of the readable beads contained the desired molecules. Regarding library screening, the Alloc group was replaced with a fluorogenic substrate (compound **77**).

## 5. Miscellaneous

Here, we review the solid-phase synthetic methodologies targeting peptidomimetics that are based on random approaches that cannot be classified within specific groups of peptidomimetics. Peptidomimetics that specifically incorporate five-membered heterocycles, such as azoles, are prepared from peptide derivatives, urea, and thiourea-containing intermediates. Table 1 summarizes examples of azole-based peptidomimetic solid-phase synthesis strategies. The peptide synthesis was predominantly conducted using classic SPPS protocols, and a variety of cyclization procedures were demonstrated.

**Scheme 14:**
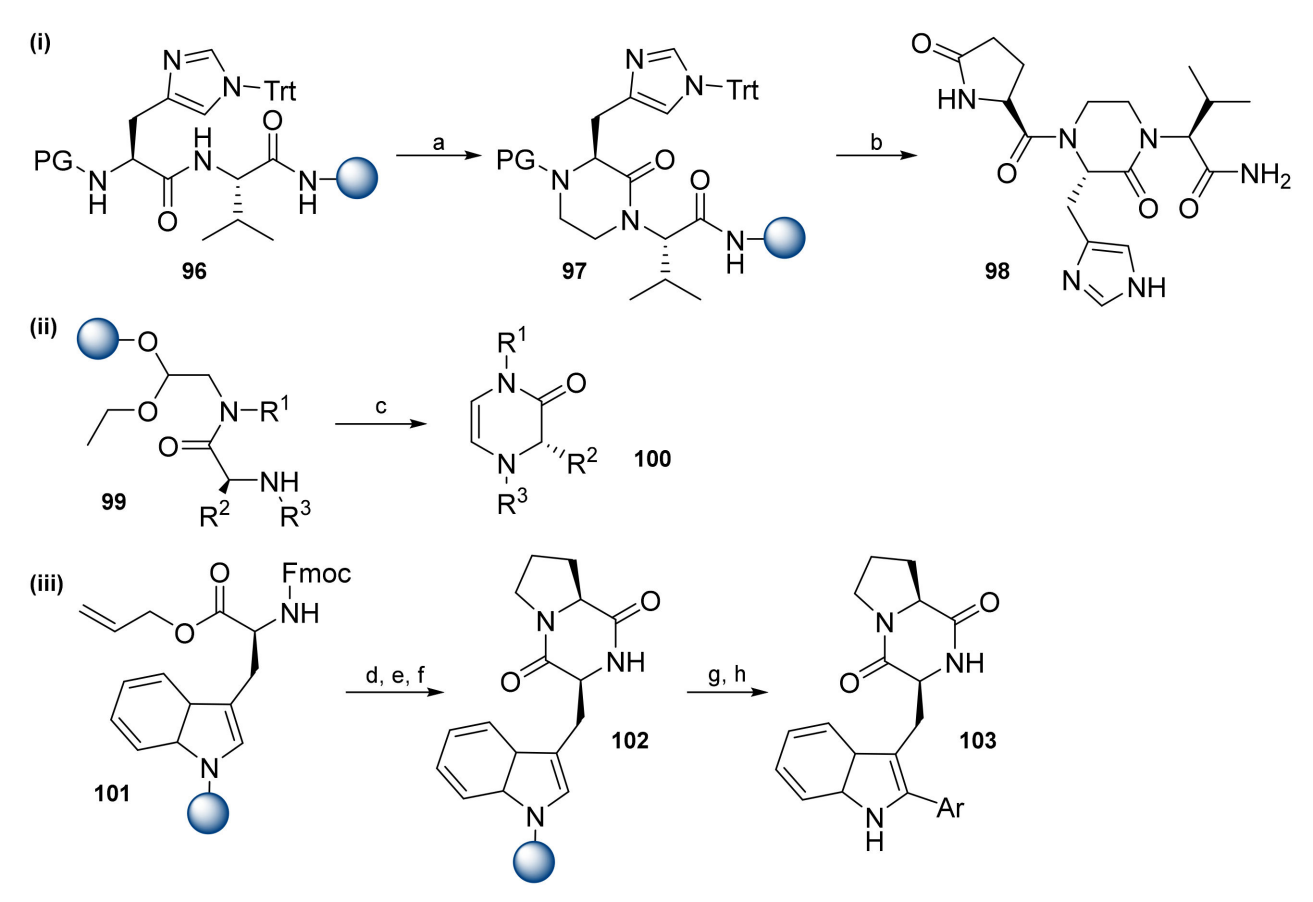
Reagents and conditions: (a) 1,2-Dibromoethane, tetramethylguanidine, DMF, 60 °C; (b) Fmoc SPPS; (c) HCO_2_H neat, 60 °C, 3 h; (d) Pd(PPh)_3_, PhSiH_3_, DCM, 2 × 30 min; (e) H-Pro-OMe HCl, PyBOP, DIPEA, DCM; (f) 20% piperidine/DMF; (g) TFA/3,5-dimethoxybenzene/DCM (0.5/0.5/9); (h) aryl halides, Pd(OAc)_2_, AgBF_4_, 2-NO_2_-Bz, PBS/DMF (1/1), MW 80 W, 80 °C, 15 min.

Teixido et al. reported on the synthesis of TRH mimetics by the replacement of the amide bond with a piperazine moiety (Scheme 14i) [58]. Resin-bound dipeptide **96** was treated with 1,2-dibromoethane in the presence of an excess of 1,1,3,3-tetramethylguanidine to afford piperazine-containing resin **97**. Fmoc SPPS provided the final compound **98**. Δ^5^-2-Oxipepazine-based peptidomimetics were synthesized using an *N*-acyliminium ion cyclization strategy (Scheme 14ii) [59]. An *N*-acyliminium ion, formed during the acidic cleavage step, generated a Δ^5^-2-oxipipepazine moiety **100** when treated with formic acid. Preciado et al. synthesized Trp-based diketopiperazine with further modification at the C2 position of Trp (Scheme 14iii) [60]. After Trp was loaded onto the resin, the allyl group of **101** was removed, and coupling with H-Pro-OMe ∙ HCl proceeded from there. Fmoc deprotection with piperidine led to spontaneous cyclization, yielding the diketopiperazine moiety. Since on-resin arylation did not produce the desired compounds, arylation was performed after cleavage from the resin. To reduce epimerization occurring during arylation, upon exposure to MW irradiation phosphate-buffered saline (PBS)/DMF (1/1) was used and provided the final compounds in good yields.

**Scheme 15:**
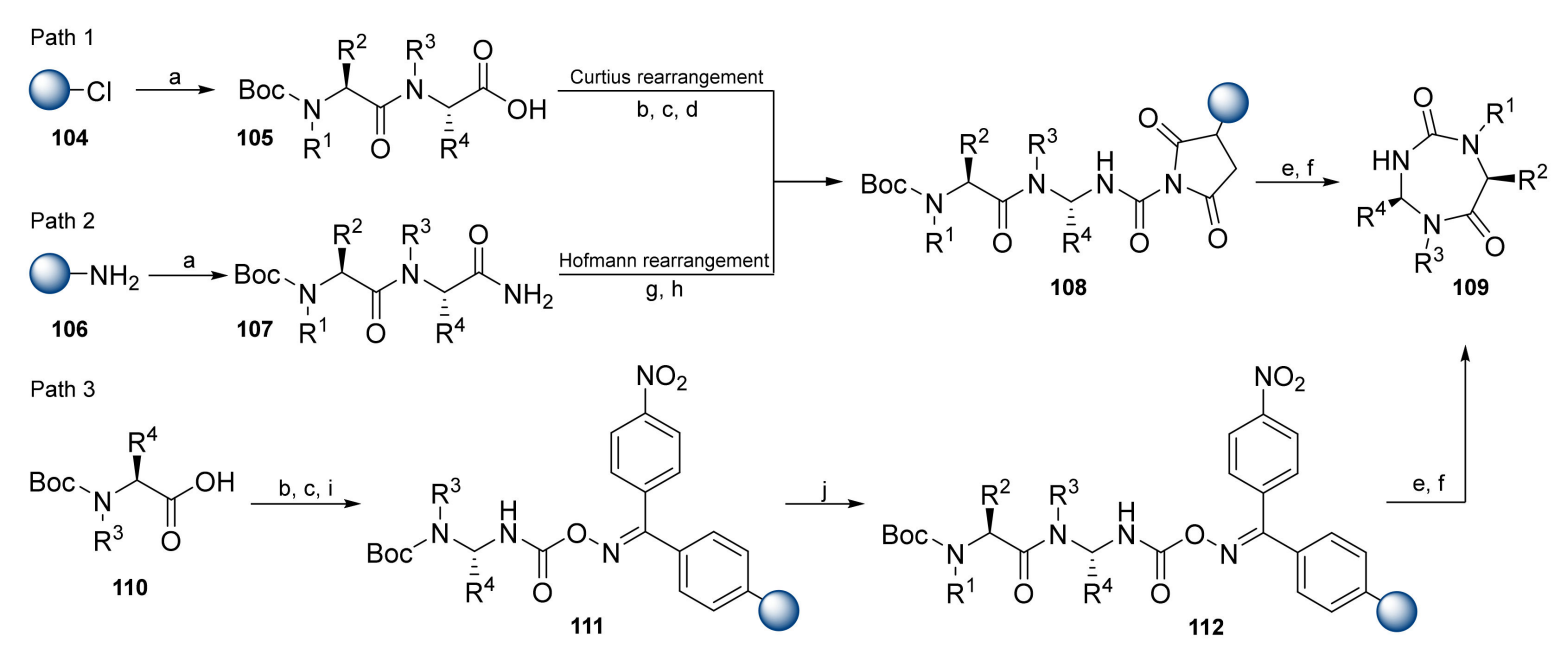
Reagents and conditions: (a) Fmoc SPPS; (b) DPPA, TEA, DMF, 30 min; (c) 70 °C, 1.5 h; (d) PS-SuOH, 70 °C, 40 min; (e) TFA/ DCM (1/3), 2 × 25 min; (g) DIPEA, DCM or THF or toluene; (g) PIFA, pyridine, THF, 1 h; (h) PS-HOSu, 5 h; (i) PS-oxime, 1 h; (j) Boc SPPS.

Boeglin et al. presented a solid-phase synthesis of dipeptide-derived 1,3,5-triazepan-2,6-diones [61,62]. The key step is the synthesis of resin-bound dipeptide intermediates (Scheme 15). According to Path 1, **105** with a carboxylic moiety was prepared on the 2-CTC resin using standard Fmoc SPPS. Upon resin loading, compound **105** was treated with diphenylphosphoryl azide (DPPA) and underwent Curtius rearrangement. Polystyrene (PS) linked with *N*-hydroxysuccinimide (HOSu) was used for the solid-phase synthesis, and as a trapping system for the dipeptidyl isocyanates. According to Path 2, compound **107,** with an amide moiety, was prepared on Sieber resin. Dipeptide **107** was treated with [bis(trifluoroacetoxy)iodo]benzene (PIFA), while pyridine and the carboxamides formed underwent Hofmann rearrangement before resin loading. Next, researchers modified the synthesis strategy by replacing the resin with PS-oxime (Kaiser oxime) resin, followed by the application of the Curtius rearrangement approach (Path 3). 1,3,5-Triazepan-2,6-diones **109** were formed via cyclorelease. Overall, Path 2 was found to be the most suitable strategy for the efficient synthesis of the 1,3,5-triazepan-2,6-dione library.

Another strategy for the peptidomimetic synthesis is the utilization of the Pictet–Spengler condensation. The approach has been successfully transferred to solid-phase synthesis to provide molecular variations including peptidomimetics, and several reviews have summarized the results [63,64,65].

## 6. Conclusions

During this review we have highlighted the solid-phase synthesis strategies for the heterocycle-based peptidomimetics. Heterocyclic compounds, such as azoles, piperazine derivatives, benzamides, and lactam derivatives, are privileged scaffolds for peptidomimetic molecules and can serve as amide bond bioisosteres. Many peptidomimetic molecules were created by target-based design and contain diverse structural features. Although standard SPPS protocols were used for general procedures, the introduction of specific units has required new approaches. Later, we expect to see emergent development of new peptidomimetics due to their undeniably attractive pharmacological properties, and these will be accompanied by new synthetic approaches. New approaches will likely include modifications to pre-existing ones. Regarding the case of solid-phase synthesis techniques, it will be important to develop new green methods to reduce the toxicity effects of the compounds, as well as to reduce the environmental burden. Currently, an excess of reagents and solvents is used for solid-phase synthesis, and some chemicals have a hazardous nature. The discovery of new eco-friendly and/or reusable solid supports, as well as solvent systems and reagents, is critical for the field. Recently, researchers have started the green transformation of solid-phase synthesis by introducing new, greener solvents [66,67], developing aqueous synthesis protocols and water-soluble reagents, and reducing the number of chemicals required [68,69]. These changes are initial yet important steps in the development of green synthetic protocols, as we expect further improvement and progress in this area. One of the promising producers of the bioactive molecules, including peptides and peptidomimetics, are host cells such as Escherichia coli or Chinese hamster ovary cells [70,71]. Genetic engineering techniques allow use of these cells as hosts for the various transformations to the novel secondary bioactive metabolites. New biosynthetic pathways are covered in several reviews [72,73].

## Figures and Tables

**Table 1:** Solid-phase synthesis strategies for the azole-based peptidomimetics.

No	Intermediate Resin	Cyclized Product	Key Strategies
1	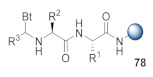	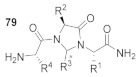	1. Formation of key intermediate **78** from resin-bound dipeptide, aldehyde, and benzotriazole; 2. Cyclization achieved via BF_3_∙Et_2_O treatment [49].
2	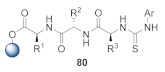	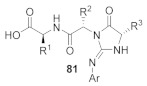	1. Formation of thiourea intermediate **80** at the *N*-terminal of the peptide; 2. Dehydrothiolation of **80** via Mukaiyama’s reagent [50].
3	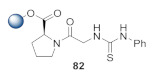	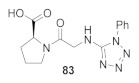	1. Incorporation of secondary amino acid into the peptide sequence; 2. Cyclization via HgCl_2_, or DIC, or Mukaiyama’s reagent [51].
4	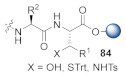	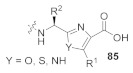	1. Oxidation and dehydrative cyclization of the Thr, Cys, Ser, diaminopropionic acid-containing dipeptides [52].
5	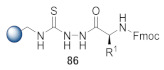	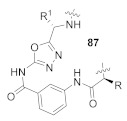	1. Backbone amide linker (BAL) strategy; 2. Thiosemicarbazide resin coupled with an amino acid to give thiourea intermediate **86**; 3. Desulfurative cyclization of **86** with *p*-TsCl and pyridine; 4. Incorporation of 3-nitrobenzoyl functionality [53].
6	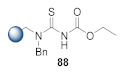	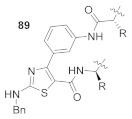	1. BAL strategy; 2. *N*-Bn-protected thiourea resin intermediate **88** was used to prevent undesired byproduct; 3. Dehydrative cyclization of **88** with 2-bromo-(3-nitrophenyl)ethenone [54].
7	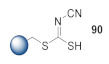	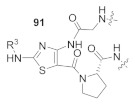	1. Traceless linker strategy; 2. Cyclization of **90** with ethyl bromoacetate to yield 1,3-thiazole [55].
8	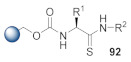	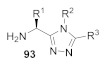	1. Cyclization of **92** with hydrazides in the presence of Hg(Ac)_2_ [56].
9	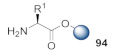	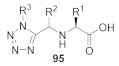	1. One-pot Ugi-azide-4CR strategy [57].

## Data Availability

Not applicable.

## References

[1] Mabonga L., Kappo A.P. (2019). Peptidomimetics: A Synthetic Tool for Inhibiting Protein–Protein Interactions in Cancer. Int. J. Pept. Res. Ther..

[2] Ivanov A.A., Khuri F.R., Fu H. (2013). Targeting protein-protein interactions as an anticancer strategy. Trends Pharmacol. Sci..

[3] Gokhale A.S., Satyanarayanajois S. (2014). Peptides and peptidomimetics as immunomodulators. Immunotherapy.

[4] Kumar M.S. (2019). Peptides and Peptidomimetics as Potential Antiobesity Agents: Overview of Current Status. Front. Nutr..

[5] Gurwitz D. (2017). Peptide Mimetics: Fast-Forward Look. Drug. Dev. Res..

[6] Qvit N., Rubin S.J.S., Urban T.J., Mochly-Rosen D., Gross E.R. (2017). Peptidomimetic therapeutics: Scientific approaches and opportunities. Drug Discov. Today.

[7] Marshall G.R., Ballante F. (2017). Limiting Assumptions in the Design of Peptidomimetics. Drug Dev. Res..

[8] Akram O.N., DeGraff D.J., Sheehan J.H., Tilley W.D., Matusik R.J., Ahn J.M., Raj G.V. (2014). Tailoring peptidomimetics for targeting protein-protein interactions. Mol. Cancer. Res..

[9] Palomo J.M. (2014). Solid-phase peptide synthesis: An overview focused on the preparation of biologically relevant peptides. RSC Adv..

[10] Made V., Els-Heindl S., Beck-Sickinger A.G. (2014). Automated solid-phase peptide synthesis to obtain therapeutic peptides. Beilstein J. Org. Chem..

[11] Garner J., Harding M.M. (2007). Design and synthesis of alpha-helical peptides and mimetics. Org. Biomol. Chem..

[12] Wilson A.J. (2015). α-Helix mimetics: Recent developments. Prog. Biophys. Mol. Biol..

[13] Azzarito V., Long K., Murphy N.S., Wilson A.J. (2013). Inhibition of alpha-helix-mediated protein-protein interactions using designed molecules. Nat. Chem..

[14] Jayatunga M.K., Thompson S., Hamilton A.D. (2014). alpha-Helix mimetics: Outwards and upwards. Bioorg. Med. Chem. Lett..

[15] Yin H., Lee G.I., Hamilton A.D., Huang Z. (2007). Alpha-Helix Mimetics in Drug Discovery. Drug Discovery Research: New Frontiers in the Post-Genomic Era.

[16] Che Y., Marshal G.R. (2008). Privileged scaffolds targeting reverse-turn and helix recognition. Expert Opin. Ther. Targets.

[17] Moon H., Lim H.S. (2015). Synthesis and screening of small-molecule alpha-helix mimetic libraries targeting protein-protein interactions. Curr. Opin. Chem. Biol..

[18] Pinto Gomes C., Metz A., Bats J.W., Gohlke H., Göbel M.W. (2012). Modular Solid-Phase Synthesis of Teroxazoles as a Class of α-Helix Mimetics. Eur. J. Org. Chem..

[19] Lee J.H., Zhang Q., Jo S., Chai S.C., Oh M., Im W., Lu H., Lim H.S. (2011). Novel pyrrolopyrimidine-based alpha-helix mimetics: Cell-permeable inhibitors of protein-protein interactions. J. Am. Chem. Soc..

[20] Yin H., Lee G.-i., Sedey K.A., Rodriguez J.M., Wang H.-G., Sebti S.M., Hamilton A.D. (2005). Terephthalamide Derivatives as Mimetics of Helical Peptides:  Disruption of the Bcl-xL/Bak Interaction. J. Am. Chem. Soc..

[21] Wrobel M., Aube J., Konig B. (2012). Parallel solid-phase synthesis of diaryltriazoles. Beilstein J. Org. Chem..

[22] Ross N.T., Katt W.P., Hamilton A.D. (2010). Synthetic mimetics of protein secondary structure domains. Philos. Trans. A Math. Phys. Eng. Sci..

[23] Yin H., Hamilton A.D., Stuart L., Schreiber T.M.K., Günther W. (2007). Protein Secondary Structure Mimetics as Modulators of Protein Protein and Protein–Ligand Interactions. Chemical Biology: From Small Molecules to Systems Biology and Drug Design, 1–3.

[24] Pelay-Gimeno M., Glas A., Koch O., Grossmann T.N. (2015). Structure-Based Design of Inhibitors of Protein-Protein Interactions: Mimicking Peptide Binding Epitopes. Angew. Chem. Int. Ed. Engl..

[25] Hirschmann R.F., Nicolaou K.C., Angeles A.R., Chen J.S., Smith III A.B. (2009). The β-d-Glucose Scaffold as a β-Turn Mimetic. Acc. Chem. Res..

[26] Metrano A.J., Abascal N.C., Mercado B.Q., Paulson E.K., Hurtley A.E., Miller S.J. (2017). Diversity of Secondary Structure in Catalytic Peptides with beta-Turn-Biased Sequences. J. Am. Chem. Soc..

[27] Feng Y., Wang Z., Jin S., Burgess B. (1998). SNAr Cyclizations to Form Cyclic Peptidomimetics of β-Turns. J. Am. Chem. Soc..

[28] Park C., Burgess K. (2001). Facile Macrocyclizations to β-Turn Mimics with Diverse Structural, Physical, and Conformational Properties. J. Comb. Chem..

[29] Li W., Burgess K. (1999). A new solid-phase linker for Suzuki coupling with concomitant macrocyclization synthesis of β-turn mimics. Tetrahedron Lett..

[30] Lee H.B., Zaccaro M.C., Pattarawarapan M., Roy S., Saragovi H.U., Burgess K. (2004). Syntheses and Activities of New C10 β-Turn Peptidomimetics. J. Org. Chem..

[31] Lee H.B., Pattarawarapan M., Roy S., Burgess K. (2003). Syntheses of second generation, 14-membered ring beta-turn mimics. Chem. Commun..

[32] Li Y., Yu Y., Giulianotti M., Houghten R.A. (2009). One-Pot High-Throughput Synthesis of β-Turn Cyclic Peptidomimetics via “Volatilizable” Supports. J. Org. Chem..

[33] Golebiowski A., Klopfenstein S.R., Chen J.J., Shao X. (2000). Solid supported high-throughput organic synthesis of peptide β-turn mimetics via tandem Petasis reaction/diketopiperazine formation. Tetrahedron Lett..

[34] Golebiowski A., Klopfenstein S.R., Shao X., Chen J.J., Colson A.-O., Grieb A.L., Russell A.F. (2000). Solid-Supported Synthesis of a Peptide β-Turn Mimetic. Org. Lett..

[35] Golebiowski A., Jozwik J., Klopfenstein S.R., Colson A.O., Grieb A.L., Russell A.F., Rastogi V.L., Diven C.F., Portlock D.E., Chen J.J. (2002). Solid-Supported Synthesis of Putative Peptide β-Turn Mimetics via Ugi Reaction for Diketopiperazine Formation. J. Comb. Chem..

[36] Kim C., Jung J., Tung T.T., Park S.B. (2016). beta-Turn mimetic-based stabilizers of protein-protein interactions for the study of the non-canonical roles of leucyl-tRNA synthetase. Chem. Sci..

[37] Kang C.W., Ranatunga S., Sarnowski M.P., Del Valle J.R. (2014). Solid-phase synthesis of tetrahydropyridazinedione-constrained peptides. Org. Lett..

[38] De Marco R., Zhao J., Greco A., Ioannone S., Gentilucci L. (2019). In-Peptide Synthesis of Imidazolidin-2-one Scaffolds, Equippable with Proteinogenic or Taggable/Linkable Side Chains, General Promoters of Unusual Secondary Structures. J. Org. Chem..

[39] Sicherl F., Cupido T., Albericio F. (2010). A novel dipeptidomimetic containing a cyclic threonine. Chem. Commun..

[40] Greco A., Tani S., De Marco R., Gentilucci L. (2014). Synthesis and Analysis of the Conformational Preferences of 5-Aminomethyloxazolidine-2,4-dione Scaffolds: First Examples of β2- and β2, 2-Homo-Freidinger Lactam Analogues. Chem. Eur. J..

[41] Bondebjerg J., Xiang Z., Bauzo R.M., Haskell-Luevano C., Meldal M. (2002). A Solid-Phase Approach to Mouse Melanocortin Receptor Agonists Derived from a Novel Thioether Cyclized Peptidomimetic Scaffold. J. Am. Chem. Soc..

[42] Smith A.B., Liu H., Okumura H., Favor D.A., Hirschmann R. (2000). Synthesis of Polypyrrolinones on Solid Support. Org. Lett..

[43] Phillips S.T., Rezac M., Abel U., Kossenjans M., Bartlett P.A. (2002). “@-Tides”:  The 1,2-Dihydro-3(6H)-pyridinone Unit as a β-Strand Mimic. J. Am. Chem. Soc..

[44] Phillips S.T., Piersanti G., Ruth M., Gubernator N., Lengerich B., Bartlett P.A. (2004). Facile Synthesis of @-Tide β-Strand Peptidomimetics: Improved Assembly in Solution and on Solid Phase. Org. Lett..

[45] Phillips S.T., Blasdel L.K., Bartlett P.A. (2005). @-Tide-Stabilized β-Hairpins. J. Org. Chem..

[46] Kang C.W., Sarnowski M.P., Ranatunga S., Wojtas L., Metcalf R.S., Guida W.C., Del Valle J.R. (2015). beta-Strand mimics based on tetrahydropyridazinedione (tpd) peptide stitching. Chem. Commun..

[47] Sarnowski M.P., Pedretty K.P., Giddings N., Woodcock H.L., Del Valle J.R. (2018). Synthesis and beta-sheet propensity of constrained N-amino peptides. Bioorg. Med. Chem..

[48] Graven A., St. Hilaire P.M., Sanderson S.J., Mottram J.C., Coombs G.H., Meldal M. (2001). Combinatorial Library of Peptide Isosters Based on Diels-Alder Reactions: Identification of Novel Inhibitors against a Recombinant Cysteine Protease from Leishmania mexicana. J. Comb. Chem..

[49] Rinnova M., Nefzi A., Houghten R.A. (2002). An efficient approach for solid-phase synthesis of peptidomimetics based on 4-imidazolidinones. Tetrahedron Lett..

[50] Gavrilyuk J.I., Evindar G., Batey R.A. (2006). Peptide−Heterocycle Hybrid Molecules: Solid-Phase Synthesis of a 400-Member Library of N-Terminal 2-Iminohydantoin Peptides. J. Comb. Chem..

[51] Gavrilyuk J.I., Evindar G., Batey R.A. (2007). Peptide-Heterocycle Hybrid Molecules : Solid-Phase-Supported Synthesis of Substituted N-Terminal 5-Aminotetrazole Peptides via Electrocyclization of Peptidic Imidoylazides. J. Comb. Chem..

[52] Biron E., Chatterjee J., Kessler H. (2006). Solid-Phase Synthesis of 1,3-Azole-Based Peptides and Peptidomimetics. Org. Lett..

[53] Abdildinova A., Yang S.J., Gong Y.D. (2018). Solid-phase parallel synthesis of 1,3,4-oxadiazole based peptidomimetic library as a potential modulator of protein-protein interactions. Tetrahedron.

[54] Cha M.-J., Abdildinova A., Gong Y.-D. (2020). Solid-phase parallel synthesis of 1,3-thiazole library adorned with dipeptidyl chains. Tetrahedron.

[55] Abdildinova A., Gong Y.D. (2021). Traceless solid-phase synthesis and β-turn propensity of 1,3-thiazole-based peptidomimetics. RSC Adv..

[56] Boeglin D., Cantel S., Heitz A., Martinez J., Fehrentz J.A. (2003). Solution and Solid-Supported Synthesis of 3,4,5-Trisubstituted 1,2,4-Triazole-Based Peptidomimetics. Org. Lett..

[57] Mendez Y., De Armas G., Perez I., Rojas T., Valdes-Tresanco M.E., Izquierdo M., Alonso Del Rivero M., Alvarez-Ginarte Y.M., Valiente P.A., Soto C. (2019). Discovery of potent and selective inhibitors of the Escherichia coli M1-aminopeptidase via multicomponent solid-phase synthesis of tetrazole-peptidomimetics. Eur. J. Med. Chem..

[58] Teixido M., Prokai-Tatrai K., Wang X., Nguyen V., Prokai L. (2007). Exploratory neuropharmacological evaluation of a conformationally constrained thyrotropin-releasing hormone analogue. Brain Res. Bull..

[59] Lee S.C., Park S.B. (2007). Practical Solid-Phase Parallel Synthesis of Δ5-2-Oxopiperazines via N-Acyliminium Ion Cyclization. J. Comb. Chem..

[60] Torres-García C., Díaz M., Blasi D., Farràs I., Fernández I., Ariza X., Farràs J., Lloyd-Williams P., Royo M., Nicolás E. (2012). Side Chain Anchoring of Tryptophan to Solid Supports Using a Dihydropyranyl Handle: Synthesis of Brevianamide F. Int. J. Pept. Res. Ther..

[61] Boeglin J., Venin C., Guichard G. (2012). Development of a practical solid-phase synthesis approach to 1,3,5-triazepan-2,6-diones. Tetrahedron.

[62] Lena G., Lallemand E., Gruner A.C., Boeglin J., Roussel S., Schaffner A.-P., Aubry A., Franetich J.-F., Mazier D., Landau I. (2006). 1,3,5-Triazepan-2,6-diones as Structurally Diverse and Conformationally Constrained Dipeptide Mimetics: Identification of Malaria Liver Stage Inhibitors from a Small Pilot Library. Chem. Eur. J..

[63] Calcaterra A., Mangiardi L., Delle Monache G., Quaglio D., Balducci S., Berardozzi S., Iazzetti A., Franzini R., Botta B., Ghirga F. (2020). The Pictet-Spengler Reaction Updates Its Habits. Molecules.

[64] Ingallina C., D’Acquarica I., Delle Monache G., Ghirga F., Quaglio D., Ghirga P., Berardozzia S., Markovic V., Botta B. (2016). The Pictet-Spengler Reaction Still on Stage. Curr. Pharm. Des..

[65] Stöckigt J., Antonchick A.P., Wu F., Waldmann H. (2011). The Pictet–Spengler Reaction in Nature and in Organic Chemistry. Angew. Chem. Int. Ed..

[66] Lawrenson S.B., Arav R., North M. (2017). The greening of peptide synthesis. Green Chem..

[67] Lawrenson S., North M., Peigneguy F., Routledge A. (2017). Greener solvents for solid-phase synthesis. Green Chem..

[68] Jad Y.E., Kumar A., El-Faham A., de la Torre B.G., Albericio F. (2019). Green Transformation of Solid-Phase Peptide Synthesis. ACS Sustain. Chem. Eng..

[69] Varnava K.G., Sarojini V. (2019). Making Solid-Phase Peptide Synthesis Greener: A Review of the Literature. Chem. Asian J..

[70] Kober L., Zehe C., Bode J. (2013). Optimized signal peptides for the development of high expressing CHO cell lines. Biotechnol. Bioeng..

[71] Gaglione R., Pane K., Dell’Olmo E., Cafaro V., Pizzo E., Olivieri G., Notomista E., Arciello A. (2019). Cost-effective production of recombinant peptides in Escherichia coli. New Biotechnol..

[72] Gopal G.J., Kumar A. (2013). Strategies for the Production of Recombinant Protein in Escherichia coli. Protein J..

[73] Hug J.J., Krug D., Müller R. (2020). Bacteria as genetically programmable producers of bioactive natural products. Nat. Rev. Chem..

